# Commentary: considerations for using the ‘Trials within Cohorts’ design in a clinical trial of an investigational medicinal product

**DOI:** 10.1186/s13063-017-2432-3

**Published:** 2018-01-08

**Authors:** Anna C. Bibby, David J. Torgerson, Samantha Leach, Helen Lewis-White, Nick A. Maskell

**Affiliations:** 1 Academic Respiratory Unit, Bristol Medical School, University of Bristol, 2nd Floor Learning & Research Building, Southmead Hospital, Bristol, BS10 5NB UK; 2 Department of Respiratory Medicine, North Bristol NHS Trust, Bristol, UK; 3 York Trials Unit, University of York, York, UK; 4 Research & Innovation, North Bristol NHS Trust, Bristol, UK

**Keywords:** Cohort multiple randomised controlled trials, Trials within cohorts, Clinical trial of an investigational medicinal product, Two-stage consent

## Abstract

**Background:**

The ‘trials within cohorts’ (TwiC) design is a pragmatic approach to randomised trials in which trial participants are randomly selected from an existing cohort. The design has multiple potential benefits, including the option of conducting multiple trials within the same cohort.

**Main text:**

To date, the TwiC design methodology been used in numerous clinical settings but has never been applied to a clinical trial of an investigational medicinal product (CTIMP). We have recently secured the necessary approvals to undertake the first CTIMP using the TwiC design. In this paper, we describe some of the considerations and modifications required to ensure such a trial is compliant with Good Clinical Practice and international clinical trials regulations.

We advocate using a two-stage consent process and using the consent stages to explicitly differentiate between trial participants and cohort participants who are providing control data. This distinction ensured compliance but had consequences with respect to costings, recruitment and the trial assessment schedule.

**Conclusion:**

We have demonstrated that it is possible to secure ethical and regulatory approval for a CTIMP TwiC. By including certain considerations at the trial design stage, we believe this pragmatic and efficient methodology could be utilised in other CTIMPs in future.

**Electronic supplementary material:**

The online version of this article (doi:10.1186/s13063-017-2432-3) contains supplementary material, which is available to authorized users.

## Background

**Fig. 1:**
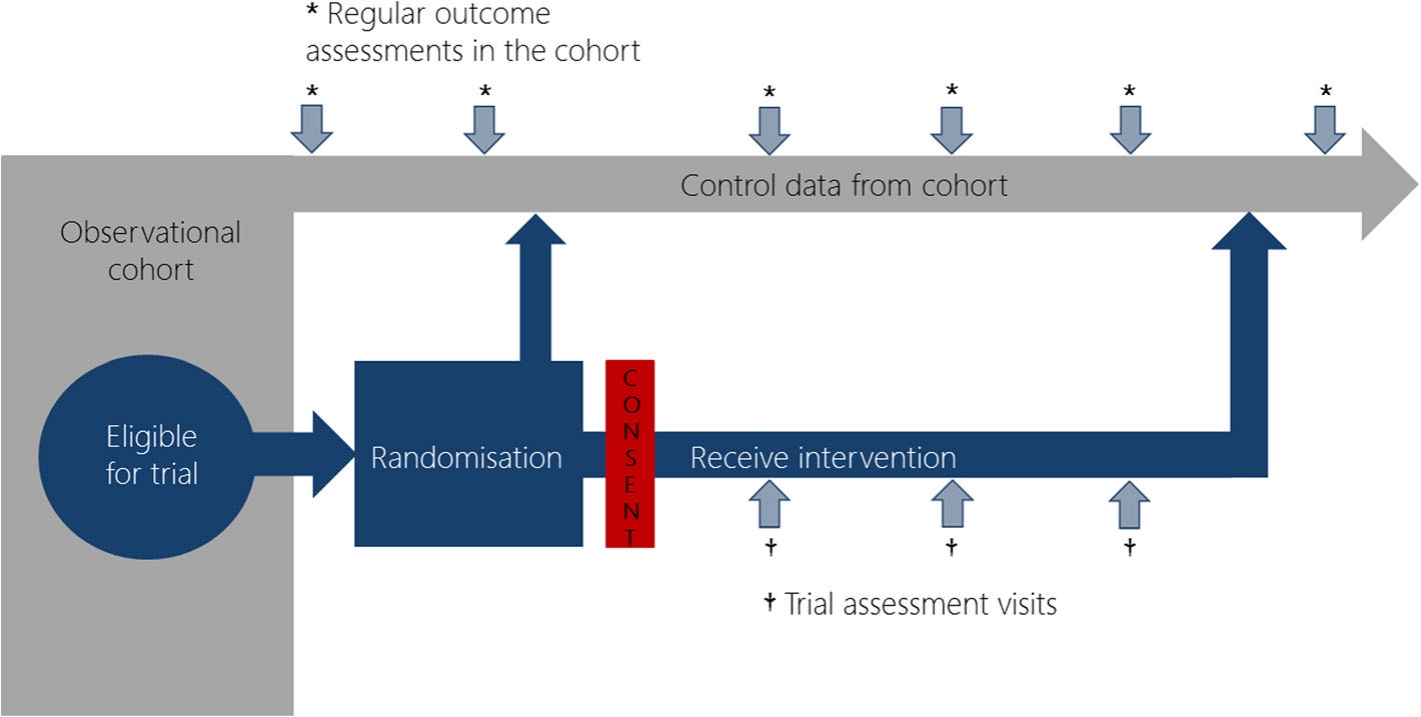
Schematic representation of the TwiC design

The trials within cohorts (TwiC) methodology, also known as the cohort multiple randomised controlled trial (cmRCT) design, is a pragmatic approach to randomised trials [1]. The method uses an existing cohort to identify eligible trial participants, a proportion of whom are then randomly selected to be offered the trial intervention. These participants are invited to discuss the intervention and, if they agree, provide consent to receive it, while participants who are eligible but not selected for the trial continue cohort follow-up. Non-selected participants are not informed about the intervention and do not provide further consent; however, their data are compared with that of trial participants and in this way they constitute a ‘control’ group (Fig. 1).

The TwiC design has many potential benefits, including efficient recruitment, reduction of cross-over, disappointment bias and attrition in the control group, and the potential to undertake multiple trials within the same cohort [1–3]. Additionally, TwiCs replicate real-life clinical care, where patients are told about interventions only when they are going to receive them and not if they are not. In this way, the methodology is highly pragmatic and can provide useful information on the effectiveness of interventions [1, 2, 4]. TwiCs may offer a solution to some of the recognised issues encountered with standard randomised clinical trials (RCTs), specifically external validity and efficiency [2, 5]. However, there has been some unease regarding the ethics of TwiCs, particularly with respect to informed consent, and diligence is required when designing a TwiC to ensure that consent processes are comprehensive yet proportionate [2, 6–9].

Since its initial description in 2010, the TwiC design has been used internationally in areas as diverse as public health, oncology, mental health and chronic disease [10–15]. To date, however, the methodology has never been used in a clinical trial of an investigational medicinal product (CTIMP). For safety reasons, CTIMPs are subject to more stringent regulations and governance than other research [16–20]; in order for a CTIMP TwiC to comply with these regulations, certain considerations are necessary.

Our research group has designed the first CTIMP TwiC in the world. The trial of intra-pleural bacterial immunotherapy in mesothelioma (‘TILT’) is a dual-centre feasibility TwiC (EudraCT no. 2016-004727-23). TILT has received Clinical Trials Authorisation (CTA) from the UK Medicines and Healthcare Regulatory Authority (MHRA) and has been approved by the local Research Ethics Committee (REC) and the UK Health Research Authority (HRA). Recruitment is scheduled to begin shortly. A summary of TILT, including the trial flow chart, is shown in Additional file 1. We wish to share our experiences of setting up TILT, for the benefit of fellow researchers planning CTIMP TwiCs.

## Main text

### Trials suitable for the TwiC design

As a highly pragmatic methodology, TwiCs can provide valuable information regarding the real-life utility and effectiveness of interventions, including investigational medicinal products. However, the methodology is less suitable for explanatory trials aiming to evaluate whether an intervention has an effect under ideal (and therefore tightly controlled) conditions. Since TwiCs rely on the use of standard care as the comparator arm, placebo-controlled trials are not possible. Trials that entail additional interventions in the control arm, beyond the remit of usual care, are also incompatible with the TwiC design, as these would require specific consent for those procedures from control participants, thus undermining one of the key tenets of TwiCs. Finally, TwiCs were designed to provide a ‘patient-centred’ consent process in which every participant is informed about, and gives consent for, the precise activities and interventions that they will undergo. Participant blinding, therefore, is not part of the design. In combination, these factors mean that, in their current format, TwiCs are not appropriate for use in early-phase CTIMPs and drug efficacy studies.

### Clinical trials regulations

Clinical research involving pharmaceutical products is guided by the International Committee for Harmonisation of Technical Requirements for Pharmaceuticals for Human Use (ICH) Statement on Good Clinical Practice (GCP) [21]. This document provides an international standard for ethical and scientific quality in research involving human participants, based on the principles set out in the Declaration of Helsinki. In the EU, this guidance has been transcribed into law in the form of European Directive 2001/20/EC, also known as the EU Clinical Trials Directive. Similar legislation has been produced by the Food & Drug Administration (FDA) in the USA [19] and other regulatory authorities in other countries. It is a legal requirement that all CTIMPs conducted in these countries adhere to the relevant regulations.

### Consent in TwiCs

The primary purpose of clinical trials legislation is to protect the safety, wellbeing and rights of trial participants. A fundamental component of this is informed consent, whereby research participants are given information about all research procedures, including any potential risks associated with those activities. Only once they have had time to consider this information will the participants be in a position to make an autonomous, informed decision regarding participation in the research.

In the context of randomised trials in participants who have capacity, the process of randomisation is a research activity and should only occur once the participant has consented to take part in the trial. Although ‘pre-randomisation’ has been used historically, notably as part of the Zelen design, it was generally considered unethical, with significant potential to damage the doctor–participant relationship [22–24]. Initially concerns were voiced that the TwiC design entailed pre-randomisation; however, proposal of a ‘staged consent’ model resolved this issue [6, 25]. With staged consent, participants provided initial consent at cohort enrolment that included agreement to undergo random selection for future trials and for use of their data as control data in the event of non-selection, without further notification. Participants selected for the intervention were then asked to provide second consent, specifically related to the trial intervention [6]. Thus, all participants provided consent for every research activity they experienced.

We adopted the staged consent method for TILT and, for additional clarity, used the consent stages to explicitly separate research processes into cohort or trial activities. Thus, screening for future TwiCs, random selection and provision of control data were designated cohort activities and covered by the cohort consent form, while investigational medicinal product (IMP) administration was a trial activity, covered by the trial consent form. Extrapolation of this approach meant that, by definition, trial participants were people who had signed the trial consent form, while everyone else, including controls, were simply participating in the cohort, albeit a comparative cohort with randomisation element.

Separating participants into trial and cohort populations in this way removed ambiguity, ensured legality and, we believe, facilitated the approvals processes. For monitoring and GCP inspection purposes, we intend to store a copy of the cohort consent form alongside the trial consent form in TILT participants’ individual files. In this way, it will be possible to reconstruct every stage of the TwiC process, with clear documentation of consent before each stage.

We believe this approach is essential for CTIMP TwiCs. According to article 4.8.10(c) of ICH GCP, participants in trials involving IMPs must be informed about the IMP and the probability of being assigned to it [21]. Without staged consent, CTIMP TwiCs fail to meet this requirement, as control participants are not informed about the IMP or the probability of being selected to receive it. However, by specifying that controls are cohort participants and that the trial population consists exclusively of participants who have signed the trial consent form (i.e. those who were selected to receive the intervention and agreed), we ensured that TILT complied with ICH GCP requirements.

However, while this approach clarified important elements of CTIMP governance, it generated complexity in other areas. These complexities are discussed below.

### Costings

If funding for the cohort and trial were secured from different sources, care is required to ensure that research costs are correctly allocated and that funders are satisfied with how their grant monies are utilised. Even though control participants are not, strictly speaking, participating in the trial, the intensity of their follow-up will be increased to match the trial assessment schedule. Since these data will assist in the analysis of trial outcomes, rather than being collected for cohort purposes, it seems appropriate to include these costs in the funding application for the trial, with a clear explanation that it will be used to cover the cost of controls in the cohort.

Another financial consideration for UK trials relates to National Institute for Health Research (NIHR) study support resources for studies included on the Clinical Research Network (CRN) Portfolio. Commensurate with the complexity of the research, a higher level of support is available for randomised trials than for observational studies. By designating controls as cohort participants rather than trial participants, we limited the level of study support that participating NHS hospitals could receive for these participants (although arguably this is an appropriate limitation since controls only undergo cohort activities). This factor should be highlighted when approaching centres to participate in the trial.

### Recruitment

As a tertiary centre involved in multiple research studies, patients are often referred to our service for consideration of clinical trials. With our standard RCTs, we encourage recruitment by publicising our trials at clinical and academic meetings around the UK, and inviting clinicians to refer willing patients to our centre for review. In doing so, local clinicians are required to inform their patients that they may be eligible for a trial and enquire whether they are willing to travel to one of the trial centres for further discussion and assessment. However, this approach was not possible with TILT, as it would have resulted in patients being made aware of the trial’s existence before enrolment in the cohort, thus rendering blinding of future control patients impossible and undermining one of the main premises of the TwiC approach.

Another consideration regarding participants from other centres was that while patients may be willing to travel a significant distance to be screened for a trial, they may be disinclined to repeat the journey if they are not selected for that trial. Consequently, if they were allocated to be a cohort-based control in a TwiC, they may decline ongoing follow-up at the trial centre, causing differential attrition.

For these reasons, recruitment to TILT was limited to the catchment area of each study centre. Screening and enrolment data collected as part of the feasibility outcomes for TILT will be analysed to determine whether this impacted on recruitment. It is possible, however, that the increased recruitment efficiency associated with the TwiC design may outweigh the effect of reduced external referrals [1].

### Study assessment schedule

CTIMPs assessment schedules are usually more intensive than observational cohorts, which tend to be more pragmatic [26]. However, to obtain meaningful comparison data in a TwiC, follow-up of cohort-based controls, there is a need to match the trial assessment schedule.

It would be impossible to design a cohort with a visit schedule that matched all potential future TwiCs. Consequently, we specified in the protocol for the cohort study that the follow-up regimen was flexible and may be altered based on clinical or research requirements. This was also mentioned in the participant information sheet for the cohort and discussed when informing potential participants about the study before enrolment. Thus, the assessment schedule of cohort participants could be adapted if they were identified as TwiC controls without violating the cohort protocol, without subjecting participants to extra assessment visits that may be considered ‘trial-related’ and, most importantly, without requiring further consent. In this way, control participants remained blinded to the existence of the TwiC, while still providing useful comparison data under circumstances covered by their original consent.

Even with flexible cohort follow-up, however, if a trial assessment schedule is particularly demanding, altering a control’s follow-up to match it may induce curiosity or anxiety and could lead to inadvertent or explicit unblinding of controls. Furthermore, it could be seen as unethical to place excessive research demands on the control population, particularly in our research setting, where participants have incurable cancer. For this reason, we designed the TILT assessment schedule to be simple and pragmatic, while remaining sufficient to generate relevant outcome data. We also ensured that the follow-up regimen was similar to routine clinical care. As such, we felt it was ethical and acceptable to alter controls’ follow-up to match the TILT schedule.

### Data collection

Although the trial team needed to appreciate the complexities of a CTIMP TwiC in order to ensure legality and compliance, the trial processes had to be as straightforward as possible in order to allow the trial to be successfully executed at multiple centres in busy clinical settings. We therefore elected to use electronic case-report forms (eCRFs) for data capture, with a common database for both cohort and trial. Branching logic was employed to ensure that the correct data fields appeared depending on whether the participant was enrolled in TILT, providing control data or simply undergoing cohort follow-up. The same programming allowed clear directions to be provided for individual participants regarding the timing of their next study visit, thus reducing confusion and the potential for error. Generation of secondary identification numbers within the database allowed easy identification of trial and control participants for data extraction and monitoring purposes.

### Ethics Committee approval and Clinical Trials Authorisation

Our experience of obtaining the necessary approvals from the Research Ethics Committee (REC) and Medicines & Healthcare Products Regulatory Agency (MHRA) was positive. A team member attended an international symposium on ethical considerations in TwiCs, at which representatives from the Health Research Authority (HRA) and MHRA gave presentations [8]. It was clear, therefore, that both organisations were familiar with the design and had given consideration to potential ethical issues. The main concern of the MHRA was that research participants were aware that they could withdraw from the study at any point and that safety monitoring requirements were in place. The HRA outlined guidance on applying a proportionate approach to consent. An independent ethics consultant was also in attendance and emphasised the importance of engaging in dialogue with RECs.

Following this symposium, the research team decided to submit the ethics applications for the cohort and the TwiC to the same REC. These submissions were made in quick succession, in January 2017 and March 2017, respectively. The same investigator (ACB) attended both REC meetings. It is our opinion that this approach facilitated the ethics approval process, as the TwiC design was discussed in length during the cohort review process, and consequently the panel were already familiar with the concept when the trial review took place. There was some discussion during the trial review meeting regarding the fact that control participants were not informed about the trial and whether this represented a form of ‘deception’. However, the clear distinction between trial participants, defined as people who had signed the trial consent form, and controls who continued to be defined as cohort participants, helped resolve this. Cohort controls were not being deceived about anything, since they had been fully informed about the potential activities involved in participating in the cohort, including altered follow-up assessment schedules and provision of control data to TwiCs.

Following submission of our application for Clinical Trials Authorisation, the MHRA requested minor amendments be made to the protocol to ensure no trial-related activities occurred before written informed trial consent was provided. In addition, they requested clarity regarding serious adverse event (AE) monitoring in control patients in the cohort. As a result, the standard operating procedure for AE monitoring for the trial was extended to apply to the cohort as well. It was recognised that this would generate extra work for research teams, but this was considered acceptable given the importance of participant safety. It also offered the additional benefit of allowing capture of late or delayed AEs that may occur after the trial had finished and participants returned to follow-up in the cohort.

## Conclusion

Our experience setting up the first CTIMP TwiC has been one of innovation and evolution. However, we have demonstrated that applying the TwiC methodology to CTIMPs is both possible and achievable, and that the necessary approvals can be obtained, governance requirements fulfilled and multiple sites initiated without undue difficulty.

We employed the two-stage consent process to explicitly separate participants into trial or cohort participants [6]. This approach ensured compliance with EU Clinical Trials Legislation and, we believe, facilitated the approvals process by ensuring our submissions to the research ethics committee and MHRA were clear and unambiguous.

We have shared our experience of the additional complexities associated with CTIMP TwiCs and hope this will be of benefit to other researchers contemplating this methodology. We await with anticipation the results of TILT, which includes feasibility outcomes and qualitative interviews exploring the experiences of participants in both the trial and the cohort.

## Additional file

Additional file 1: TILT summary and trial flow chart. (DOCX 103 kb)

## Abbreviations

cmRCT: Cohort multiple randomised controlled trial
CRN: Clinical Research Network
CTIMP: Clinical trial of an investigational medicinal product
EU: European Union
GCP: Good Clinical Practice
HRA: Health Research Authority (UK)
ICH: International Committee for Harmonisation of Technical Requirements for Pharmaceuticals for Human Use
MHRA: Medicines and Healthcare Products Regulatory Agency (UK)
NIHR: National Institute for Health Research
REC: Research Ethics Committee
TILT: Trial of intra-pleural bacterial immunotherapy in mesothelioma
TwiC: Trials within cohorts
