# Supplementary material for: Commentary: considerations for using the ‘Trials within Cohorts’ design in a clinical trial of an investigational medicinal product
Source: Trials. 2018 Jan 8;19:18. doi: 10.1186/s13063-017-2432-3 (PMC5759253; doi:10.1186/s13063-017-2432-3)
Supplement: Additional file 1: — TILT summary and trial flow chart. (DOCX 103 kb) [file 13063_2017_2432_MOESM1_ESM.docx]

**Appendix A – TILT summary and trial flowchart**

1. BACKGROUND

Malignant pleural mesothelioma (MPM) is an aggressive cancer of the lung lining. It is incurable, with median survival less than 1 year from diagnosis(1-7). Chemotherapy extends survival by 2.8 months, but is not suitable for everyone(1, 2, 4). The UK has the highest mortality rates for MPM worldwide, with 2535 people dying from it in 2012(2). Incidence is rising, and is predicted to peak in the next decade(1-3).

MPM is an attractive target for immunotherapy as the tumour evades the immune response that usually inhibits tumour growth(8-11). MPM suppresses populations of protective T cells in the pleura^10-13^. The ability to overcome this is associated with longer survival(12-15), and drugs that stimulate T cell activity are currently undergoing clinical trials(16-18).

T cells are stimulated by bacteria. Observational studies report enhanced survival in patients with lung cancer and pleural infection(19-22). A similar phenomenon has been reported in a series of patients with pleural infection and malignant effusions, in which patients with MPM and pleural infection lived over a year longer than non-infected patients(23). Historically, intra-pleural bacteria have been used as pleurodesis agents, with some demonstrating concomitant, anti-tumour activity(24-34). Attenuated bacterial agents have been shown to control pleural fluid and prolong survival in malignant pleural effusions secondary to lung cancer(35-39). In MPM, mice injected with staphylococcus enterotoxin demonstrated florid T cell responses in association with reduced tumour growth(40).

This research will investigate OK432, a penicillin and heat-treated, lyophilized mixture of group A Streptococcus pyogenes. OK432 induced lymphocyte-mediated tumour killing in pleural fluid in vitro, controlled malignant pleural effusions in vivo and improved survival in lung cancer(35-37).

This project will investigate intra-pleural OK432 in MPM using an innovative trial design, the Trial within a Cohort (TwiC)(41). Participants will be recruited from an existing observational cohort, consisting of regular clinical and radiological monitoring (the ASSESS-meso study). From ASSESS-meso, 25 eligible participants will be randomly selected to receive OK432, delivered intra-pleurally as a single dose via an indwelling catheter. Outcome data will be compared with control participants from ASSESS-meso. Qualitative interview will be undertaken to assess acceptability of the methodology to participants.

1. AIM

The aim of this research is to explore the role of intra-pleural OK432 in people with MPM, and assess the feasibility and acceptability of the TwiC methodology. The study aims to answer the question “Is it feasible to undertake a TwiC of intra-pleural OK432 in MPM and is it acceptable to participants and relatives?” If feasibility is demonstrated, results will inform the design of a full-scale TwiC of intra-pleural OK432 in MPM.

1. STUDY DESIGN

This is a feasibility study for a single-blind, randomised trial of intra-pleural OK432 in people with mesothelioma, based on the trial within a cohort design.

1. POPULATION & SETTING

The study will take place at two centres: North Bristol NHS Trust and Oxford University Hospitals Trust. Participants will be patients with MPM who are participating in the ASSESS-meso study and who have given their consent to be screened and randomly selected for clinical trials and who meet the TILT eligibility criteria.

- 1. INCLUSION CRITERIA

To participate in TILT patients must meet all of the following inclusion criteria:

- Histological or cytological diagnosis of MPM
- Enrolled in ASSESS-meso and has given consent to be considered for and be randomly selected for future trials
- IPC in situ that has drained more than 50ml of fluid on previous 3 drainages

OR willing to have an IPC and has a pleural effusion suitable for IPC insertion

- No chemotherapy in preceding 4 weeks and none planned in subsequent 4 weeks
- Performance status ≤2, or PS 3 and felt clinically suitable for trial
- Predicted survival ≥12 weeks from enrolment
- Able to give written informed consent & meet trial requirements
  1. EXCLUSION CRITERIA

To be eligible for TILT, participants must have none of the following exclusion criteria:

- No IPC in situ, and has contra-indication to IPC insertion
- Clinico-radiological diagnosis of MPM
- Trapped lung with <50% pleural apposition on x-ray
- Moderately heavy or heavily loculated pleural effusion
- Known immunodeficiency or immuno-suppressive medication
- Intercurrent infection (pleural or elsewhere) or clinical signs of sepsis
- Known sensitivity or allergy to OK432 or penicillin
- Previous treatment with immunotherapy
- Currently enrolled in any other interventional clinical trial
- Brain metastases or CNS involvement of MPM
- Pregnancy or lactation, current or planned during the study period
- Age <18
- Any other factor that, in the opinion of the Chief Investigator, would mean participation in the study would be contraindicated

1. TRIAL PROCESSES

Patients with mesothelioma will be invited to participate in a longitudinal observational cohort study, consisting of pleural fluid monitoring, patient-reported outcome measurements, chest radiography (CXR) and blood tests (the ASSESS-meso study). From this cohort, 45 eligible participants for TILT will be identified, 25 of whom will be randomly selected to receive a single dose of intra-pleural OK432. Randomisation will be undertaken by a centralised, concealed computerised system, and will occur on a 1:1 basis, with minimisation by tumour sub-type and WHO/ECOG performance status (PS). Participants selected to receive OK432 will receive it via an indwelling pleural catheter (IPC), on an unblinded basis. Participants who decline to receive OK432 following random selection will continue to undergo observational follow up, and their data will be collected as part of the feasibility outcomes.

Participants who are not selected to receive OK432 will continue longitudinal assessments as part of ASSESS-meso, blinded to the existence of the OK432 arm. This is consistent with real-life clinical care, wherein patients are told about treatments as and when they are going to receive them.

1. OUTCOME MEASUREMENTS

The primary outcome measure is feasibility, which will be assessed based on the following criteria:

- Screening, eligibility and recruitment rates to TILT
- Time taken to recruit participants
- Acceptance rates for intra-pleural OK432 following random selection
- Attrition rates
- Data completeness rates
- Collection of data on participants who decline any element of the trial
- Qualitative interviews with participants and their relatives to explore the acceptability of trial processes.

Secondary outcome measures will collect exploratory data on the clinical efficacy of OK432. They will include:

- Tumour response, based on CT appearances at baseline and 3 months, assessed using modified RECIST(42)
- Overall survival (OS), defined as time between date of diagnosis with MPM to date of death, censored at the end of the trial (EoT)
- Progression-free survival rates (PFSR) at 3 months
- Patient-reported chest pain and breathlessness, measured on visual analogue scales (VAS)
- Patient-reported quality of life, measured using the EQ-5D-5L health questionnaire
- Pleurodesis rates, defined as pleural fluid drainage of less than 50ml on 3 consecutive occasions, with <25% opacification on CXR or <250ml pleural fluid on thoracic ultrasound scanning (TUS)
- Biomarker response assessed using serial serum mesothelin levels.

Participants will be followed up until death or End of Trial (EoT), whichever is sooner.

- 1. **Trial flowchart**

See page 4 for the trial flowchart.

**TILT Trial**

Intra-pleural OK432

Consents to receive

OK432?

**End of cohort follow up**

**Final visit**

Day 84

**Follow up 1**

Day 14

**Intervention**

Day 0-7

**Screening**

**Day 0**

**ASSESS-meso cohort**

Randomly

selected to receive

OK432?

Becomes eligible for TILT

Y

N

Y

N

**Follow up 2**

Day 35

**References**

1. Peake MD BP, Woolhouse I, et al on behalf of the National Lung Cancer Audit Project Team. National Lung Cancer Audit Report 2014, mesothelioma. Report for the period 2008–2012 <http://www.hscic.gov.uk/media/15038/Mesothelioma-audit/pdf/EMBARGOEDTO120914_NLCA_Meso_Report_final.pdf>: Health & Social Care Information Centre; 2014 [

2. Beckett P, Edwards J, Fennell D, Hubbard R, Woolhouse I, Peake MD. Demographics, management and survival of patients with malignant pleural mesothelioma in the National Lung Cancer Audit in England and Wales. Lung Cancer. 2015;88(3):344-8.

3. Hodgson J, McElvenny D, Darnton A, Price M, Peto J. The expected burden of mesothelioma mortality in Great Britain from 2002 to 2050. British Journal of Cancer. 2005;92(3):587.

4. Vogelzang NJ, Rusthoven JJ, Symanowski J, Denham C, Kaukel E, Ruffie P, et al. Phase III study of pemetrexed in combination with cisplatin versus cisplatin alone in patients with malignant pleural mesothelioma. J Clin Oncol. 2003;21(14):2636-44.

5. Yates D, Corrin B, Stidolph P, Browne K. Malignant mesothelioma in south east England: clinicopathological experience of 272 cases. Thorax. 1997;52(6):507-12.

6. Herndon JE, Green MR, Chahinian AP, Corson JM, Suzuki Y, Vogelzang NJ. Factors Predictive of Survival Among 337 Patients With Mesothelioma Treated Between 1984 and 1994 by the Cancer and Leukemia Group B. Chest. 1998;113(3):723-31.

7. Ribak J, Selikoff IJ. Survival of asbestos insulation workers with mesothelioma. British Journal of Industrial Medicine. 1992;49(10):732-5.

8. Boon T, van der Bruggen P. Human tumor antigens recognized by T lymphocytes. The Journal of Experimental Medicine. 1996;183(3):725-9.

9. Dunn GP, Old LJ, Schreiber RD. The immunobiology of cancer immunosurveillance and immunoediting. Immunity. 2004;21(2):137-48.

10. Lew F, Tsang P, Holland JF, Warner N, Selikoff IJ, Bekesi JG. High frequency of immune dysfunctions in asbestos workers and in patients with malignant mesothelioma. J Clin Immunol. 1986;6(3):225-33.

11. Hegmans JP, Hemmes A, Hammad H, Boon L, Hoogsteden HC, Lambrecht BN. Mesothelioma environment comprises cytokines and T-regulatory cells that suppress immune responses. European Respiratory Journal. 2006;27(6):1086-95.

12. Leigh RA, Webster I. Lymphocytic infiltration of pleural mesothelioma and its significance for survival. SAMJ, S Afr med j. 1982;61(26):1007-9.

13. Suzuki K, Kadota K, Sima CS, Sadelain M, Rusch VW, Travis WD, et al. Chronic inflammation in tumor stroma is an independent predictor of prolonged survival in epithelioid malignant pleural mesothelioma patients. Cancer Immunol Immunother. 2011;60(12):1721-8.

14. Anraku M, Cunningham KS, Yun Z, Tsao MS, Zhang L, Keshavjee S, et al. Impact of tumor-infiltrating T cells on survival in patients with malignant pleural mesothelioma. J Thorac Cardiovasc Surg. 2008;135(4):823-9.

15. Yamada N, Oizumi S, Kikuchi E, Shinagawa N, Konishi-Sakakibara J, Ishimine A, et al. CD8+ tumor-infiltrating lymphocytes predict favorable prognosis in malignant pleural mesothelioma after resection. Cancer Immunol Immunother. 2010;59(10):1543-9.

16. Calabro L, Morra A, Fonsatti E, Cutaia O, Amato G, Giannarelli D, et al. Tremelimumab for patients with chemotherapy-resistant advanced malignant mesothelioma: an open-label, single-arm, phase 2 trial. Lancet Oncology. 2013;14(11):1104-11.

17. Calabrò L, Morra A, Fonsatti E, Cutaia O, Fazio C, Annesi D, et al. Efficacy and safety of an intensified schedule of tremelimumab for chemotherapy-resistant malignant mesothelioma: an open-label, single-arm, phase 2 study. Lancet Respiratory Medicine. 2015;3(4):301-9.

18. Kindler HL, Karrison T, Khattri A, Zuo Z, Sulai N, Rose B, et al., editors. P2.08-010 Phase II Study of the Anti-PD-1 Antibody Pembrolizumab in Patients with Malignant Mesothelioma. 16th World Conference on Lung Cancer; 2015; Denver, USA: Journal of Thoracic Oncology.

19. Sensenig D, Rossi N, Ehrenhaft J. Results of the surgical treatment of bronchogenic carcinoma. Surgery, Gynecology & Obstetrics. 1963;116:279-84.

20. Takita H. Effect of postoperative empyema on survival of patients with bronchogenic carcinoma. J Thorac Cardiovasc Surg. 1970;59(5):642.

21. Ruckdeschel JC, Codish SD, Stranahan A, McKneally MF. Postoperative empyema improves survival in lung cancer: documentation and analysis of a natural experiment. New England Journal of Medicine. 1972;287(20):1013-7.

22. Virkkula L, Kostiainen S. Postpneumonectomy empyema in pulmonary carcinoma patients. Scandinavian Journal of Thoracic and Cardiovascular Surgery. 1970;4(3):267-70.

23. Bibby AC, Clive AO, Slade GC, Morley AJ, Fallon J, Psallidas I, et al. Survival in Patients With Malignant Pleural Effusions Who Developed Pleural Infection: A Retrospective Case Review From Six UK Centers. Chest. 2015;148(1):235-41.

24. McKneally M, Maver C, Kausel H, Alley R. Regional immunotherapy with intrapleural BCG for lung cancer. J Thorac Cardiovasc Surg. 1976;72(3):333-8.

25. McKneally M, Maver C, Lininger L, Kausel H, McIlduff J, Older T, et al. Four-year follow-up on the Albany experience with intrapleural BCG in lung cancer. J Thorac Cardiovasc Surg. 1981;81(4):485-92.

26. Ruckdeschel JC, McKneally MF, Baxter DH, DeVore C, Kellar S, Killam D, et al. Regional immunotherapy has a detrimental effect on the response to combined irradiation and chemotherapy in locally advanced non-small cell bronchogenic carcinoma. Cancer Immunol Immunother. 1981;11(4):277-82.

27. Bakker W, Nijhuis-Heddes J, van der Velde E. Post-operative intrapleural BCG in lung cancer: a 5-year follow-up report. Cancer Immunol Immunother. 1986;22(2):155-9.

28. Bakker W, Nijhuis-Heddes JM, de la Rivière AB, Dijkman JH. Complications of postoperative intrapleural BCG in lung cancer. Annals of Thoracic Surgery. 1982;33(3):267-72.

29. Ostrowski M, Priestman T, Houston R, Martin W. A randomized trial of intracavitary bleomycin and Corynebacterium parvum in the control of malignant pleural effusions. Radiotherapy and Oncology. 1989;14(1):19-26.

30. Millar J, Hunter A, Horne N. Intrapleural immunotherapy with Corynebacterium parvum in recurrent malignant pleural effusions. Thorax. 1980;35(11):856-8.

31. Webb H, Oaten S, Pike C. Treatment of malignant ascitic and pleural effusion with Corynebacterium parvum. British Medical Journal. 1978;1(6109):338-40.

32. Casali A, Gionfra T, Rinaldi M, Tonachella R, Tropea F, Venturo I, et al. Treatment of malignant pleural effusions with intracavitary Corynebacterium parvum. Cancer. 1988;62(4):806-11.

33. McLeod D, Calverley P, Millar J, Horne N. Further experience of Corynebacterium parvum in malignant pleural effusion. Thorax. 1985;40(7):515-8.

34. Rossi GA, Felletti R, Balbi B, Sacco O, Cosulich E, Risso A, et al. Symptomatic Treatment of Recurrent Malignant Pleural Effusions with Intrapleurally Administered Corynebacterium parvum: Clinical Response Is Not Associated with Evidence of Enhancement of Local Cellular-Mediated Immunity 1–3. American Review of Respiratory Disease. 1987;135(4):885-90.

35. Uchida A, Micksche M. Lysis of fresh human tumor cells by autologous peripheral blood lymphocytes and pleural effusion lymphocytes activated by OK432. Journal of the National Cancer Institute. 1983;71(4):673-80.

36. Luhr KT, Yang PC, Kuo SH, Chang DB, Yu CJ, Lee LN. Comparison of OK‐432 and mitomycin C pleurodesis for malignant pleural effusion caused by lung cancer. A randomized trial. Cancer. 1992;69(3):674-9.

37. Sakamoto J, Teramukai S, Watanabe Y, Hayata Y, Okayasu T, Nakazato H, et al. Meta-analysis of adjuvant immunochemotherapy using OK-432 in patients with resected non-small-cell lung cancer. Journal of Immunotherapy. 2001;24(3):250-6.

38. Masuno T, Kishimoto S, Ogura T, Honma T, Niitani H, Fukuoka M, et al. A comparative trial of LC9018 plus doxorubicin and doxorubicin alone for the treatment of malignant pleural effusion secondary to lung cancer. Cancer. 1991;68(7):1495-500.

39. Ren S, Terman DS, Bohach G, Silvers A, Hansen C, Colt H, et al. Intrapleural staphylococcal superantigen induces resolution of malignant pleural effusions and a survival benefit in non-small cell lung cancer. Chest. 2004;126(5):1529-39.

40. Lansley SM, della Vergiliana V, Julius F, Cleaver AL, Ren SH, Segal A, et al. A commercially available preparation of Staphylococcus aureus bio‐products potently inhibits tumour growth in a murine model of mesothelioma. Respirology. 2014;19(7):1025-33.

41. Relton C, Torgerson D, O’Cathain A, Nicholl J. Rethinking pragmatic randomised controlled trials: introducing the “cohort multiple randomised controlled trial” design. British Medical Journal. 2010;340:c1066.

42. Byrne MJ, Nowak AK. Modified RECIST criteria for assessment of response in malignant pleural mesothelioma. Annals of Oncology. 2004;15(2):257-60.
